# A Ka-Band Omnidirectional Metamaterial-Inspired Antenna for Sensing Applications

**DOI:** 10.3390/s25113545

**Published:** 2025-06-04

**Authors:** Khan Md. Zobayer Hassan, Nantakan Wongkasem, Heinrich Foltz

**Affiliations:** 1 Department of Electrical, Computer and Systems Engineering, Case Western Reserve University, Cleveland, OH 44106, USA; kxh606@case.edu; 2 Department of Electrical and Computer Engineering, College of Engineering and Computer Science, The University of Texas Rio Grande Valley, Edinburg, TX 78539, USA; heinrich.foltz@utrgv.edu

**Keywords:** metamaterial, omnidirectional, antenna, sensor, 5G, Ka-band, air pollution, toxic metal, particles

## Abstract

A Ka-Band, 26.5–40 GHz, omnidirectional metamaterial-inspired antenna was designed, built, and tested to develop a simple printed compact (10.3 mm × 10.3 mm × 0.0787 mm) multiple-point sensor for air pollution monitoring. This Ka-band antenna generated a dual band at 27.49–29.74 GHz and 33.0–34.34 GHz. The VSWR values within the two bands are less than 1.5. The radiation and total efficiency are 97% and 92% in the first band and they are both 96% in the second band. The maximum gain is between 3.26 and 5.50 dBi and between 5.09 and 6.52 dBi in the first and second bands, respectively. The dual band is the key to enhancing the sensor’s detection accuracy. This Omni MTM-inspired antenna/sensor can effectively detect toxic and neurotoxic metal particles, i.e., lead, zinc, copper, and nickel, in evidently polluted living environments, such as factory/industrial environments, with different particle/mass concentrations. This sensor can be adapted to detect metal pollutants in different environments, such as water or other fluid-based matrices, and can also be applied to long-range communication repeaters and 5G harvesting energy devices, to name a few.

## 1. Introduction

“Omni-sensory” describes the ubiquity of sensors, whereby sensing capabilities are present everywhere. It has been implemented to monitor multiple environmental parameters, e.g., temperature, humidity, moisture content, dew point, pressure, energy usage, liquid flow rates, sound, vibration, air particle count, weather [1,2], or some specific data, for instance, tissue strain [3], visual data mining [4], and more. The sensing elements are typically designed based on the parameters being measured.

Microwave sensors [5,6], which operate in the frequency range of 300 MHz to 300 GHz, have been used to detect particle sizes through scattering effects [7,8,9]. Microwave sensor frequency and particle size interact significantly. The operating frequency directly influences the sensitivity and accuracy of particle size measurement. For example, a 2.4 GHz microwave sensor can accurately detect particles in the 70–200 μm range [10] or, on average, 0.2% of the operating wavelength. 

Expanding omni-sensing parameters and possibilities to a higher microwave frequency range can enhance sensitivity performance to spot smaller particle sizes; we, therefore, propose a highly efficient, low-profile, wideband metamaterial-inspired antenna sensor operating in the Ka-band, 26.5–40 GHz. This sensor is specifically designed to effectively detect toxic and neurotoxic metal particles, i.e., lead (Pb), zinc (Zn), copper (Cu), and nickel (Ni) [11], in hypothetically polluted living environments, factory/industrial, wherein particle diameters can vary between 0.1 µm and 10 µm. Our case studies show that the antenna’s broad frequency band and high radiation efficiency significantly boost the sensitivity and detection distance of omnidirectional sensors.

The sensor can be adapted to detect metal pollutants in different environments, such as water or other fluid-based matrices, if the system can effectively exhibit the Mie resonance, displaying the absorption and scattering properties of the small spherical metal particles (pollutants) embedded in a dielectric host. Moreover, effective medium approximations (EMAs) for metal–dielectric composites can be implemented to calculate the overall properties (e.g., effective dielectric constant) based on the properties of their components (metal particles and a dielectric host). Maxwell–Garnett and Bruggeman are common EMAs used for this purpose. The effective properties of a composite material change with varying parameters like particle size, volume fraction, and the properties of the individual components.

## 2. Omni Metamaterial-Inspired Antenna Design

A Ka-band, low-profile, omnidirectional metamaterial (MTM) antenna [12,13] was designed to have a dual band with high gain and efficiency. An omnidirectional radiation pattern and a dual band were achieved by accurately designing the metamaterial [14,15,16,17,18,19,20,21,22,23] radiating patch and the ground plane.

### 2.1. Metamaterial Structure Design

**Figure 1:**
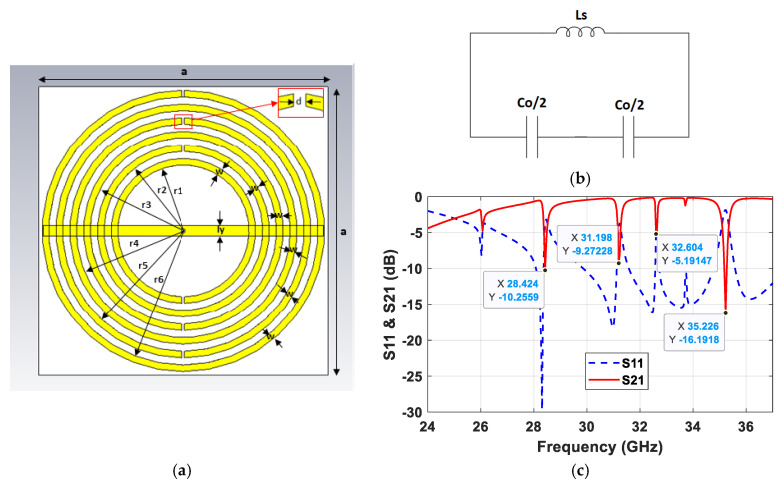
(**a**) Proposed MTM structure with (**b**) its equivalent circuit of two adjacent rings and its (**c**) S-parameters.

The proposed metamaterial structure consists of six concentric split-ring resonators (SRR), as shown in Figure 1a. The rings were designed using 0.03 mm (1 oz) thick copper trace and placed on a 10.3 × 10.3 mm^2^ Rogers RT5880/Duroid substrate ($\varepsilon_{r}$ = 2.2) with a thickness of 0.787 mm. The dimensions of the MTM structure are as follows: a = 10.3 mm, r_1_ = 2.36 mm, r_2_ = 2.85 mm, r_3_ = 3.34 mm, r_4_ = 3.83 mm, r_5_ = 4.32 mm, r_6_ = 4.81 mm, w = 0.23 mm, d = 0.1 mm, and l_y_ = 0.35 mm.

As illustrated in Figure 1b, the equivalent circuit of the SRR can be modeled with self-inductance *L_s_* associated with each ring and capacitance *C_o_*/2 for each SRR half, where *C_o_* = 2π*r_o_C_pul_* [24]. The specific equations of *L_s_* and *C_pul_* can be found in reference [25]. The resonant frequency of the structure is given by *f_o_* = (*L_s_C_s_*)^−1/2^/2π, where *C_s_ = C_o_*/4 [26], which is the series capacitance of the upper and lower halves of the SRR.

The MTM structure was analyzed as a periodic structure with plane-wave excitation using CST Microwave Studio. The transmission coefficient, S_21_, and reflection coefficient, S_11_, are shown in Figure 1c. There are four transmission bands, centered at 28.42, 31.20, 32.60, and 35.23 GHz. These bands are implemented in the antenna’s radiating patch design in the next section. Other material parameters, i.e., the permeability, permittivity, and refractive index of the MTM, can be extracted, if needed, using the S-parameters [27].

### 2.2. Metamaterial Antenna Design

#### 2.2.1. Antenna Geometry

**Figure 2:**
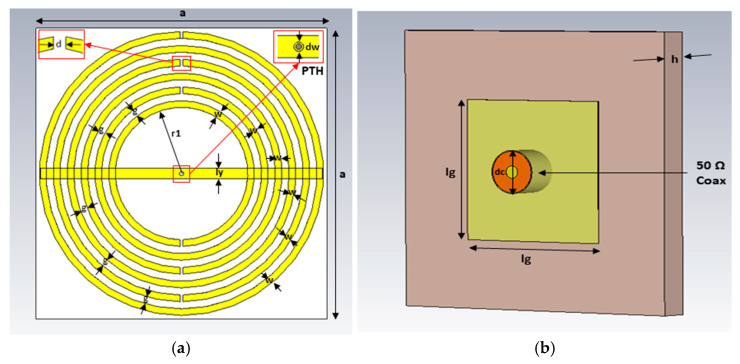
MTM antenna geometry: (**a**) MTM radiating patch (front view) and (**b**) ground plane with probe feed (back view).

The MTM structure, designed to have a response in the Ka-band, is used as a radiating patch. The antenna is fed underneath at the center. Figure 2 shows the geometry of the proposed antenna. The MTM radiating patch consists of six slitted concentric rings and a connecting rectangular bar. The antenna, 10.3 × 10.3 × 0.787 mm^3^, was designed and built using a two-sided 1 oz copper Rogers RT5880/Duroid ($\varepsilon_{r}$ = 2.2, tan δ = 0.0009). To establish an electrical connection between the radiating patch and the connector probe, a plated through-hole (denoted as dw) of 0.15 mm diameter was designed at the geometric center of the metamaterial structure, connecting to the 50-ohm coaxial cable.

#### 2.2.2. Antenna Design Optimization

**Figure 3:**
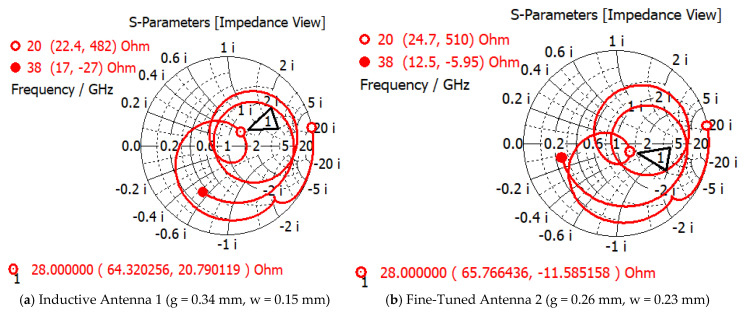
Smith chart of (**a**) inductive and (**b**) fine-tuned antennas.

To match the antenna input impedance to 50-ohm port impedance, the net reactance of the structure should be zero at the frequency of interest to ensure the perfect resonance condition. The inductance is created by the metal strips, depending on the length, width, geometry, and arrangement of the traces. The capacitance can be manipulated by gaps, slits, or slots in the antenna structure. Two distinct sets of values of width (w) and gap (g) for the radiating patch, designated as antenna 1 (g = 0.34 mm, w = 0.15 mm) and antenna 2 (g = 0.26 mm, w = 0.23 mm), were chosen for this analysis. Antenna 1 presents inductive behavior, as the 28 GHz operating frequency point lies on the upper half of the chart, shown in Figure 3a. Adding capacitance to the structure controls the operating point to align more toward the center of the chart. This can be achieved by either increasing the surface area of the rings (increasing the ring width, w, or reducing the gaps, g, between rings, or a combination of both, as illustrated for antenna 2 (Figure 3b).

The rectangular metal strip has a significant impact on EM coupling on the antenna structure. By varying the width of the strip (ly), good control over the coupling and hence impedance matching can be achieved, i.e., ly = 0.35 mm in this design.

**Figure 4:**
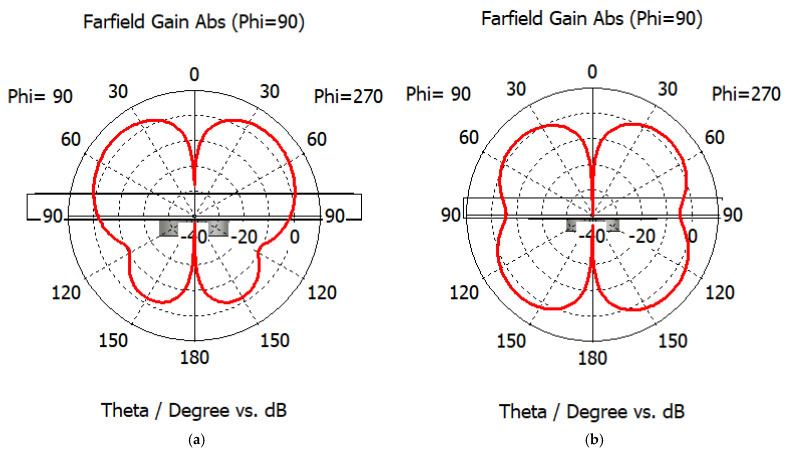
Radiation pattern of antennas of (**a**) lg = 10.3 mm and (**b**) lg = 5.2 mm.

Figure 4 illustrates the effect of changing the edge length, lg, of the squared ground plane of the antenna to create an omnidirectional radiation pattern. When the bottom of the substrate is covered entirely by the ground plane (lg = 10.3 mm), most of the radiation in the downward direction is blocked, giving the pattern a more directional shape (Figure 4a). As the dimension of the plane is reduced, the radiation pattern leans more toward an omnidirectional shape, and the optimum case was found for lg = 5.2 mm (Figure 4b). Further reduction in the size of the ground plane results in a narrower waist in the θ = 90° direction.

#### 2.2.3. Final Design and Results

Table 1 lists the antenna optimization parameters used for fabrication.

The prototype sensor/antenna was built using silver, which has the highest conductivity of all metals. For mass production with cost consideration, antennas/sensors, fabricated using copper, will also provide a similar response, if operated between 300 MHz and 300 GHz. However, to prevent surface oxidation, the Cu surface should be coated with self-assembled monolayers (SAMs), e.g., Benzotriazole.

**Figure 5:**
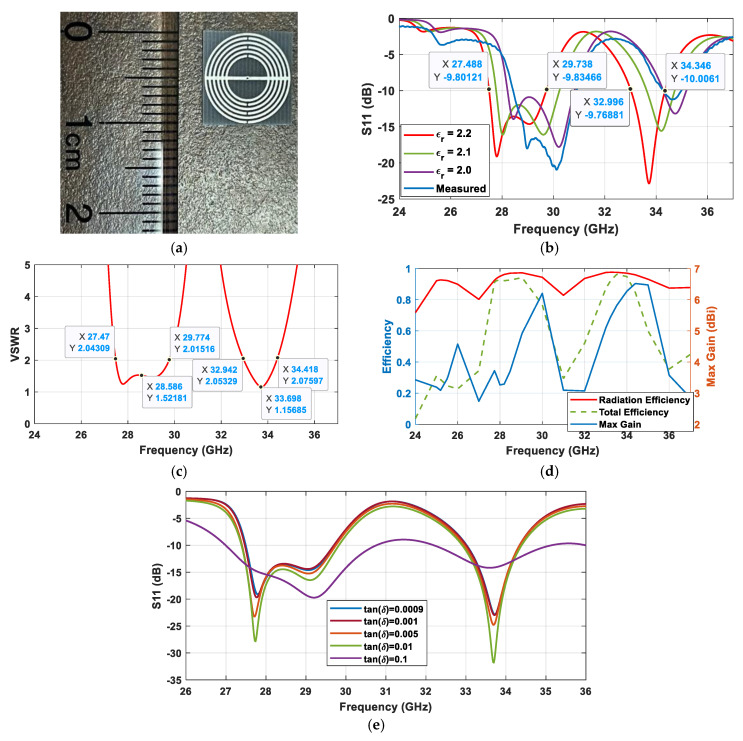
(**a**) Fabricated MTM-inspired antenna, (**b**) its S_11_ with different substrate dielectric constants, (**c**) VSWR, (**d**) radiation efficiency, total efficiency, and maximum gain, and (**e**) S_11_ with different substrate loss tangents.

Figure 5a presents the fabricated prototype of the proposed antenna. A 2.4 mm male connector was connected at the center of the ground plane. The antenna exhibits two resonance bands at 27.49–29.74 and 33.0–34.34 GHz, which are intentionally designed for multiple point detection for sensing applications, shown in Figure 5b. The measured S_11_ has an offset of 1 GHz to the right, peaking at 30 GHz with a bandwidth of 2.48 GHz between 28.42 and 30.90 GHz. This offset is attributed to fabrication tolerance, impedance mismatch due to improper soldering of the connector, a change/decrease in the dielectric constant of the substrate due to high heat exposure while soldering ($\varepsilon_{r}$ is inversely proportional to the temperature), or imperfection in the substrate material itself. As confirmed and shown in Figure 5b, S_11_ shifts to the right as the dielectric constant of the substrate decreases. The VSWR values within the two bands are less than 1.5, as illustrated in Figure 5c. The radiation and total efficiency are 97% and 92%, respectively, in the first band, and they are both 96% in the second band, presented in Figure 5d. The maximum gain is between 3.26–5.50 dBi and 5.09–6.52 dBi in the first and second bands, respectively. If by any chance the substrate loss tangent is higher from the fabrication cause, the S_11_ performance will be lower, i.e., less negative. The disorder effect study is presented in Figure 5e.

**Figure 6:**
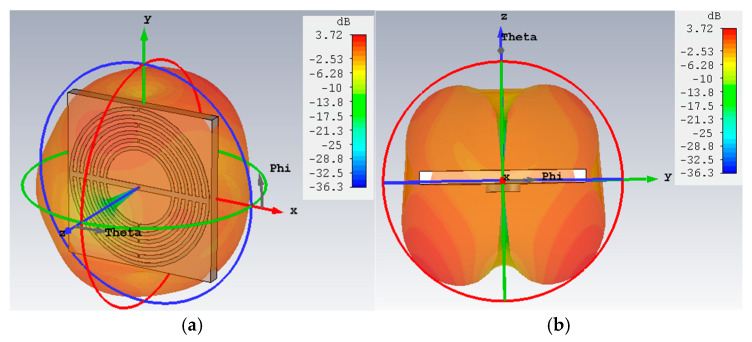
Three-dimensional far-field radiation pattern of the proposed antenna at 28 GHz: (**a**) perspective and (**b**) side view.

The simulated 3D far-field radiation pattern at 28 GHz is shown in Figure 6, presenting the omnidirectional pattern, matching with the 2D polar plot previously shown in Figure 4b.

**Figure 7:**
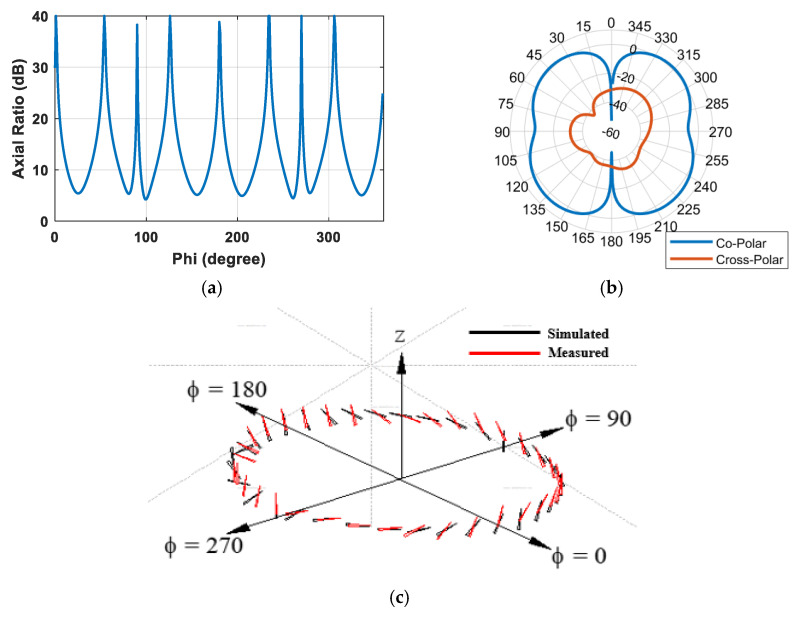
(**a**) Axial ratio, (**b**) co- and cross-components, and (**c**) polarization vector diagram of the proposed antenna.

The antenna’s axial ratio and co- and cross-components and the polarization vector diagram of the proposed antenna are presented in Figure 7. These parameters can be further investigated to advance other potential applications.

Table 2 presents the antenna performance comparison among the published 28 GHz antennas. Compared with the other Omni-antennas listed and bold in Table 2, the proposed antenna is more compact, with higher gain and higher radiation efficiency. It is also important to note that the proposed omni-antenna generates dual bands within the Ka-band, which is the key to enhancing the sensor’s detection sensitivity, which will be discussed in the next section.

**Figure 8:**
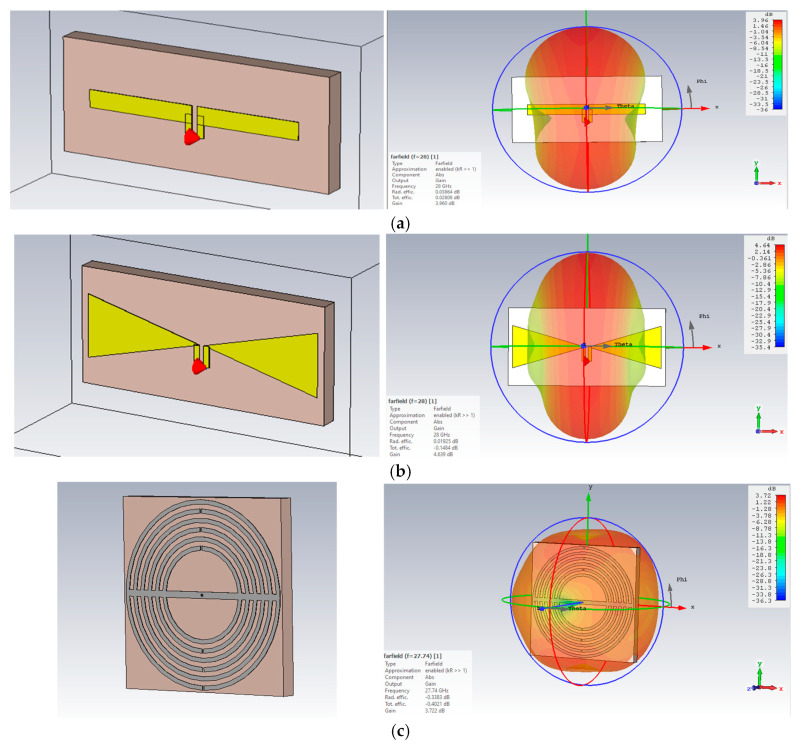
Printed (**a**) dipole, (**b**) bowtie, and (**c**) MTM-inspired antennas with their far-field radiation patterns.

**Figure 9:**
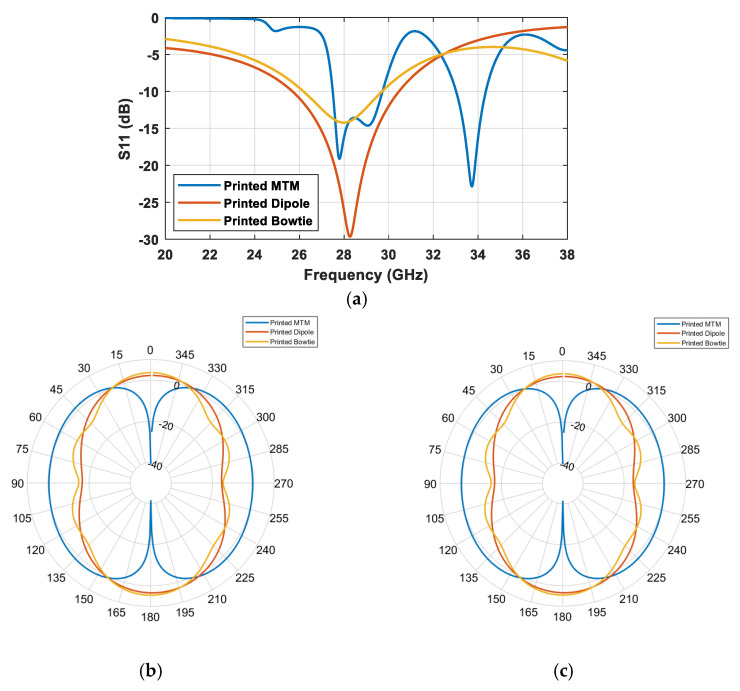
Comparison of (**a**) S_11_ and polar plots in (**b**) the X-Z plane and (**c**) the Y-Z plane of the printed MTM, dipole, and bowtie antennas.

Figure 8 illustrates two conventional omnidirectional printed antennas and our proposed MTM antenna with their 3D radiation patterns: dipole (Figure 8a), bowtie (Figure 8b), and the proposed MTM (Figure 8c). The comparisons of their S_11_ and polar X-Z and Y-Z plane plots are presented in Figure 9.

**Figure 10:**
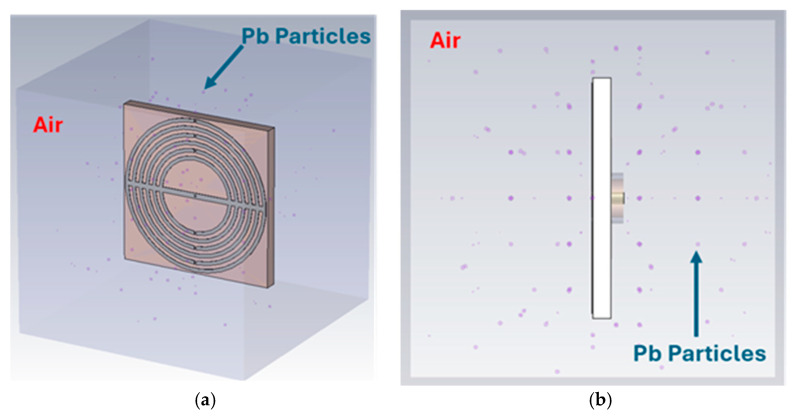
Omni MTM antenna/sensor testing in a Pb-polluted environment: (**a**) perspective and (**b**) side view.

As stated earlier, the proposed MTM-inspired antenna exhibits two bands within the range of interest. Furthermore, the radiation pattern of the MTM antenna in the XZ plane indicates better omni-radiating coverage, resulting in better sensitivity handling. The S_11_ and polar plots are presented in Figure 10.

## 3. Sensing Performance Demonstration

The proposed Ka-band Omni MTM-inspired antenna was tested to sense particulate air pollution (PM) in hypothetically polluted living environments, such as factory/industrial. Figure 10 presents the setting in which the MTM antenna/sensor is placed in the studied environment, wherein lead particles of diameters ranging from 0.1 to 10 µm are randomly placed in the atmosphere.

**Figure 11:**
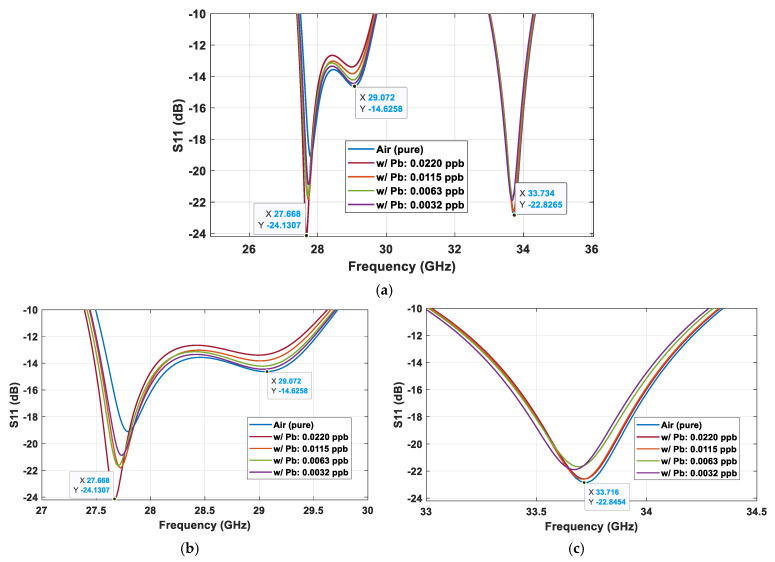
S_11_ parameters when the Omni MTM sensor was tested in Pb-polluted environments with different concentrations at different resonance frequencies: (**a**) 25–36 GHz, (**b**) 27–30 GHz, and 33–34.5 GHz.

Figure 11 presents S_11_ parameters from four case studies, with lead pollutants of four different concentration levels, i.e., 0.022, 0.0115, 0.0063, and 0.0032 ppb (parts per billion) in terms of volume, which are 42, 22, 12, and 6 million particles/m^3^ (or 126.3, 63.15, and 31.58, and 15.79 g/m3, respectively), were used to test the Omni MTM sensor’s performance. The occurrence of the Pb particles can be clearly differentiated by the three resonances at 27.67 GHz, 29.07 GHz, and 33.73 GHz, as shown in Figure 11a. At the first resonance (Figure 11b), S_11_ from the highest Pb concentration occurs at the lowest frequency with the lowest values. S11 shifts to a higher frequency as the concentration is reduced toward the pure air environment. The S_11_ behavior is opposite at the second and the third resonances at 29.07 GHz and 33.73 GHz. The sensitivity is lower when the particle concentration is reduced. At 0.0032 ppb, the response nears that of pure air yet is still distinguishable. The detectable threshold of this setting is at 0.0032 ppb. These multiple-point detections significantly boost the sensor’s accuracy.

**Figure 12:**
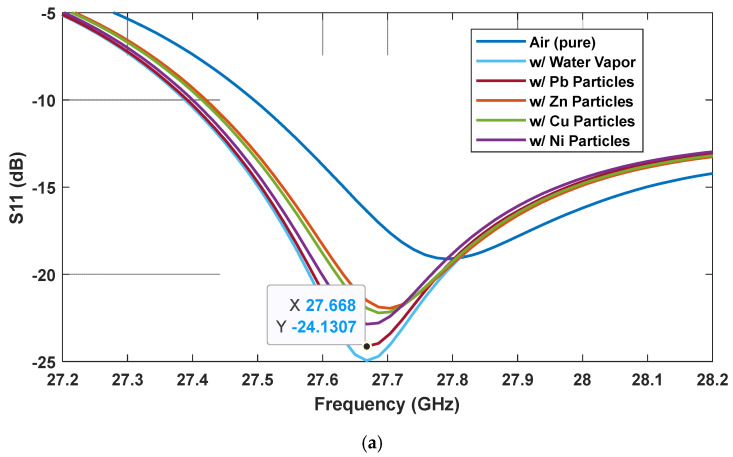

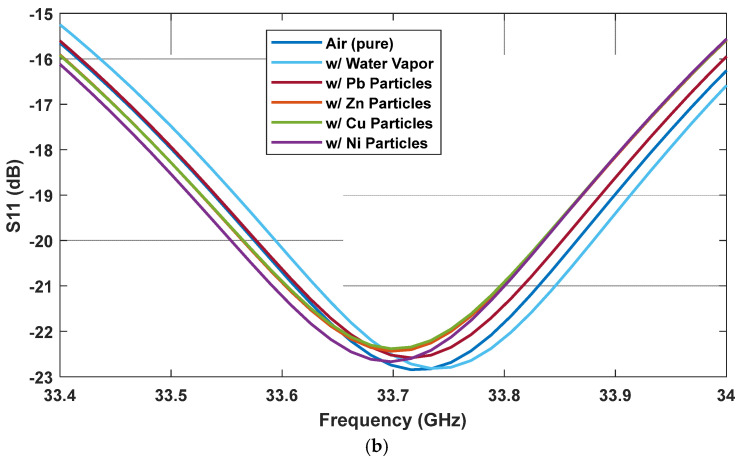
S_11_ parameters when the Omni MTM sensor was tested in Pb-, Zn-, Cu-, and Ni-polluted environments at the two resonances: (**a**) 27.2–28.2 GHz and (**b**) 33.4–34 GHz.

The Omni MTM-inspired MTM sensor was also tested with other toxic metal particles, i.e., zinc (Zn), copper (Cu), and nickel (Ni). Table 3 presents the mass concentration of the four tested metal particles. Figure 12 illustrates the S_11_ response of the Omni MTM sensor when undergoing testing in an environment with Pb, Zn, Cu, and Ni particle concentrations of 0.022 ppb. At the first resonance, presented in Figure 12a, all toxic metal-polluted cases are detected at the same frequency, 27.67 GHz, but at different intensities. The metal types can be differentiated by their S_11_ values, from high negative to low negative: Pb, Ni, Cu, and Zn. For the second resonance, shown in Figure 12b, the difference is observed through the frequency shift, from low to high frequency: Ni, Cu, Zn, and Pb. The S_11_ results show the detection capabilities, differentiating the environments in which the toxic metal particles are polluted.

## 4. Conclusions

A Ka-band omnidirectional metamaterial-inspired antenna with a dual band was designed, with the purpose of implementing it as a microwave sensor for air pollution, focusing on toxic metal particles, i.e., lead, zinc, copper, and nickel in polluted living environments, such as factory/industrial. The radiation pattern of the MTM antenna indicates exceptional omni-radiating coverage, resulting in better sensitivity handling. Our case studies have shown that this Omni MTM-inspired antenna/sensor can effectively detect toxic metal particles with different particle/mass concentrations. The dual band enhances the sensor’s detection sensitivity. Additional bands can boost its sensing performance. Mixed toxic metal environmental pollutants can be further investigated.

## Figures and Tables

**Table 1:** Optimized design parameters.

Parameter	Value	Parameter	Value
r1	2.36 mm	Dw	0.15 mm
W	0.23 mm	Ly	0.35 mm
G	0.26 mm	Lg	5.25 mm
D	0.1 mm	A	10.3 mm

**Table 2:** Performance comparison of the 28 GHz printed antenna.

Ref.	Band (GHz)	Size (mm^3^)	Radiation Pattern	Number of Bands	Rad. Eff. (%)	Gain (dBi)
[28]	27.74–28.45	15 × 15 × 0.6	Directional	Single	97	8.8
[29]	24.35–31.13	6.2 × 7 × 0.2	Directional	Single	86	8.2
**[30]**	**26.65–29.2**	**14 × 12 × 0.3**	**Omni**	**Single**	**78**	**1.7**
[22]	26–31	11 × 31 × N/A	Directional	Single	N/A	10
[15]	24.25–27.5	30 × 30 × 0.5	Directional	Single	N/A	7.4
**This work**	**27.49–29.72**	**10.3 × 10.3 × 0.7**	**Omni**	**Dual**	**97**	**5.5**

**Table 3:** Mass concentration of lead, zinc, copper, and nickel of 0.022 ppb.

Metal Particles	Mass Concentration (g/m^3^)
Pb	126.30
Zn	99.83
Cu	79.53
Ni	99.12

## Data Availability

The original contributions presented in this study are included in the article. Further inquiries can be directed to the corresponding author.
